# Neutrophil to lymphocyte ratio and platelet to lymphocyte ratio, are they markers of COVID-19 severity or old age and frailty? A comparison of two distinct cohorts

**DOI:** 10.3389/fmed.2023.1222692

**Published:** 2023-07-27

**Authors:** Yochai Levy, Estela Derazne, Alex Shilovsky, Dana Kagansky, Alex Derkath, Victor Chepelev, Evelina Mazurez, Ilia Stambler, Nadya Kagansky

**Affiliations:** ^1^Rabin Medical Center, Beilinson Hospital, Petah-Tikva, Israel; ^2^Sackler School of Medicine, Tel Aviv, Israel; ^3^Shmuel Harofe Geriatric Medical Center, Be'er Ya'akov, Israel; ^4^Shamir Medical Center, Zerifin, Israel

**Keywords:** nursing homes, geriatric, frailty, old age, COVID-19, neutrophil to lymphocyte ratio, platelet to lymphocyte ratio

## Abstract

The neutrophil-lymphocyte ratio (NLR) and platelet-lymphocyte ratio (PLR) are simple markers of systemic inflammatory responses. It has been previously suggested that they can predict COVID-19 severity. Age and frailty may also influence their values. This study aimed to evaluate the impact of COVID-19 severity versus age and frailty on NLR and PLR values. This was a retrospective, observational two cohorts’ comparative study. The first cohort is comprised of patents positive for SARS-CoV-2, with mild or asymptomatic disease, admitted to designated COVID-19 departments in a large geriatric medical center (GMC). The second included patients with COVID-19 admitted to designated COVID-19 departments in a large general hospital for symptomatic disease from March 2020 to March 2021. We compared baseline characteristics including comorbidities and chronic medications, disease symptoms, laboratory tests and compared the NLR and PLR between the two groups. The 177 patients admitted to the COVID-designated department in the GMC were over three decades older than the 289 COVID-19 patients admitted to the general hospital care (HC). They had substantially more comorbidities and chronic medications. All common disease symptoms were significantly more common in the HC group. Almost two thirds of the GMC patients remained asymptomatic compared to 2.1% in the HC group. Inflammatory markers, such as CRP and LDH, were significantly higher in the HC group. The NLR and PLR were both significantly higher in the GMC cohort comprised of older frailer patients with milder disease. NLR and PLR seem to be affected more by age and frailty than COVID-19 severity.

## Introduction

The novel coronavirus SARS-CoV-2 has caused a life changing pandemic, threatening millions of people worldwide. The clinical presentation of coronavirus disease (COVID-19) ranges from asymptomatic or mild disease to severe pneumonia, respiratory failure, and death (1). The neutrophil to lymphocyte ratio (NLR) and platelet to lymphocyte ratio (PLR) are simple inflammatory markers easily calculated from routine complete blood count. Prior studies suggested they have a prognostic role in various medical conditions such as malignancies (2–4), cardiovascular diseases (5), urinary tract infections (6, 7) influenza (7) and more.

Several studies have found NLR and PLR to be prognostic factors of COVID-19 severity with higher values representing a more severe disease and poorer prognosis (8–16). Frailty and age may also influence NLR and PLR reference values (17–21) and are also significant risk factors for severe COVID-19 (22–25). This has the potential to cause significant bias in the studies of NLR and PLR in COVID-19 patients. In Israel, a policy of routine screening for SARS-CoV-2 among nursing home residents was implemented early during the pandemic. Most dependent, asymptomatic residents positive for SARS-CoV-2 were isolated and managed in designated COVID-19 departments of skilled nursing homes or geriatric hospitals. Patients with disease considered moderate or severe were mostly admitted to hospital care (26). This policy created two diverse cohorts, one of older frailer adults with mild disease and the other of hospitalized patients with more severe disease. The two cohorts represent opposite poles in COVID-19 severity and in baselines characteristics of age, frailty and comorbidities. In this study, we aimed to evaluate which of these poles has a more significant impact on NLR and PLR in COVID-19 patients.

## Materials and methods

This was a retrospective, observational two cohorts’ comparative study. The first cohort was comprised of patents who were positive for SARS-CoV-2, with mild or asymptomatic disease, admitted to designated COVID-19 departments in a large skilled geriatric medical center (GMC). The second cohort included patients admitted to the affiliated large hospital for symptomatic COVID-19 from March 2020 to March 2021. All SARS-CoV-2 tests were performed using real-time reverse-transcription polymerase chain reaction (RT-PCR) analysis of throat swabs.

Inclusion criteria were the admission to a designated COVID-19 department, either in the geriatric hospital or in the affiliated hospital. Data was retrieved from electronic medical records (EMRs) and included age, gender, demographic variables, comorbidities, medications, laboratory tests on admission, especially inflammatory markers and disease symptoms, for both cohorts. We aimed to examine their association with the NLR and PLR.

## Statistical analysis

Sample size calculation: Considering *α* = 0.05 and 1−*β* = 0.8, NLR population mean difference 2.5 and pooled SD = 8, a sample size of 162 patients is required. For PLR population mean difference of 45 and pooled SD = 150, a sample size of 176 patients is required.

We used Chi Square Test, or Fisher’s Exact Test (2*2 tables) to compare categorical characteristics between the GMC and HC groups. Because the continuous variables were not normally distributed, we presented the results as median (25th – 75th percentiles) and used the Mann–Whitney test to compare the 2 groups.

Statistical analysis was performed using IBM SPSS Statistics for Windows, version 29 Armonk, NY: IBM Corp. Two-sided value of *p* ≤ 0.05 was considered statistically significant.

## Results

Between March 2020 to March 2021 data was collected from 177 patients admitted to the COVID-designated department in the geriatric medical center (GMC) and 289 COVID-19 patients admitted to the general hospital care (HC). Baseline characteristics are described in Table 1. The cohorts were significantly different. The GMC cohort was comprised of older adults in need of continuous supervision and help in their basic activities of daily living (27) due to limitations in physical or mental status. Patients in the GMC group were, on average, over three decades older than in the HC group. Only 38% of GMC patients were men compared to 54% men in the HC cohort. The majority of the GMC patients (53.4%) were nursing homes residents, compared to 6.9% in the HC group. And 80% of GMC patients had more than five chronic diseases, compared to 8.3% in the HC group. All the patients in the GMC cohort were frail with a Clinical Frailty Scale (CFS) (28) score ≥ 5, whereas frailty was not assessed in the HC group.

**Table 1:** Baseline characteristics.

	GMC		HC		*p*
	*N*	Median	*N*	Median	
Age	177	85 (77–91)	289	52 (43–58)	<0.001
BMI	48	23.9 (20.5–26)	273	27.78 (24.69–31.56)	<0.001
*Sex*					0.001
Male	68	38.4	158	54.7	
Female	109	61.6	131	45.3	
*Residence*					<0.001
Home	82	46.6	269	93.1	
Nursing Home	94	53.4	20	6.9	
*Total number of diseases*					<0.001
0	0	0	99	34.3	
1	1	0.6	49	17	
2	3	1.7	59	20.4	
3	14	7.9	33	11.4	
4	16	9	25	8.7	
≥5	143	80.8	24	8.3	
CHF	40	22.6	10	3.5	<0.001
CRF	39	22	11	3.8	<0.001
Dementia	61	34.5	19	6.6	<0.001
Depression	28	15.8	9	3.1	<0.001
Asthma_COPD	16	9	24	8.3	0.865
CVA	38	21.5	2	0.7	<0.001
DM	87	49.2	69	23.9	<0.001
HTN	143	80.8	77	26.6	<0.001
Hypertriglyceridemia	74	41.8	1	0.3	<0.001
Coronary Disease	53	29.9	23	8	<0.001
Hyperlipidemia	75	42.4	68	23.5	<0.001
*Total number of medications*					<0.001
0	0	0	131	45.3	
1–3	8	4.5	74	25.6	
4–6	43	24.3	49	17	
>6	126	71.2	35	12.1	
Ace_Arb_inh	77	43.5	53	18.3	<0.001
B-blockers	84	47.5	39	13.5	<0.001
NSAID’S	0	0	6	2.1	0.087
Insulin	35	19.8	27	9.3	0.002
Ca-blockers	63	35.6	27	9.3	<0.001
Vit.D	64	36.2	11	3.8	<0.001
Antiplatelets	56	31.6	42	14.5	<0.001
Eltroxin	37	20.9	12	4.2	<0.001
Anticoagulants	77	43.5	15	5.2	<0.001
Antipsychotics	64	36.2	21	7.3	<0.001
Antidepressants	50	28.2	33	11.4	<0.001

Risk factors for severe COVID-19 were significantly more prevalent in the GMC group, including hypertension, diabetes, chronic cardiovascular diseases, chronic kidney disease and malignancies. BMI and chronic respiratory diseases did not differ between the groups. Patients in the GMC group also suffered significantly more from dementia and depression (34.5% vs. 6.6 and 15.8% vs. 3.1% respectively). The majority of patients in the GMC group had polypharmacy of over 6 chronic medications, compared to only 12% in the HC group. Antihypertensive medications, beta blockers, antiplatelet anticoagulants and insulin treatment were all more prevalent in the GMC group, representing the higher disease burden. Vitamin D supplements, antipsychotics and antidepressants were also more prevalent in the GMC group. All common disease symptoms were significantly more frequent in the HC group (Table 2). Fever was the most common symptom and was found in 68.9% of the HC group compared to 10.7% in the GMC group. Cough, fatigue and dyspnea were also among the prevalent symptoms, all of which were significantly more prevalent in the HC group. Almost two thirds of the GMC patients remained asymptomatic compared to 2.1% in the HC group. The routine admission blood test results are presented in Table 3. White blood cell count, neutrophils and platelets were within the normal value range, but significantly higher in the GMC group. Lymphocytes were in the lower normal range and were higher in the HC group. Inflammatory markers, such as CRP and LDH, were also significantly higher in the HC group. The median admission in the COVID department was 15 days (median 10–20) in the GMC group versus 6 days (median 3–6) in the HC group.

**Table 2:** Symptoms.

	GMC		HC		*p*
	*N*	%	*N*	%	
Anosmia	0	0.0	13	4.5	0.002
Diarrhea	1	0.6	31	10.7	<0.001
Fatigue	7	4.0	153	52.9	<0.001
Headache	0	0.0	45	15.6	<0.001
Fever	19	10.7	199	68.9	<0.001
Cough	14	7.9	146	50.5	<0.001
Anxiety	1	0.6	4	1.4	0.654
Delirium	2	1.1	1	0.3	0.560
Dyspnea	44	24.9	134	46.4	<0.001
Syncope	1	0.6	15	5.2	0.007
Instability	25	14.1	22	7.6	0.027
Chest pain	2	1.1	44	15.2	<0.001
Change of appetite	3	1.7	49	17.0	<0.001
No symptoms	114	64.4	6	2.1	<0.001

**Table 3:** Blood tests results.

		GMC		HC		*p*
		*N*	Median (25th–75th)	N	Median (25th–75th)	
WBC	10^3^/μl	177	7.8 (5.7–10.1)	289	5.7 (4.2–7.4)	<0.001
Neutr %	%	177	72 (62.8–80.6)	289	70.2 (62.9–78.1)	0.114
Neutr# TN	10^3^/μl	177	5.53 (3.73–7.91)	289	3.9 (2.7–5.3)	<0.001
Lymph%	%	177	17.9 (10.9–25.8)	289	20.2 (14–25.9)	0.025
Lymph# TN	10^3^/μl	177	1.29 (0.9–1.7)	289	1 (0.8–1.5)	<0.001
PLT	10^3^/μl	177	239 (194–324)	289	185 (143–238)	<0.001
RDW	%	177	14.4 (13.4–15.9)	287	13.7 (13.1–14.5)	<0.001
Hb	g/dl	177	12.3 (10.6–13.6)	289	13.4 (12.5–14.4)	<0.001
Albumin	g/dl	171	3.69 (3.34–4.06)	261	3.8 (3.5–4.1)	0.073
CRP	mg/dl	155	26 (11–56)	283	53.13 (16.91–111.96)	<0.001
Creatinine	mg/dl	176	0.92 (0.72–1.2)	286	0.76 (0.63–0.92)	<0.001
LDH	U/l	118	334 (272–416)	260	527 (414–677.5)	<0.001
Urea	mg/dl	176	50 (35–74.5)	288	25.6 (19.85–31.7)	<0.001

**Figure 1:**
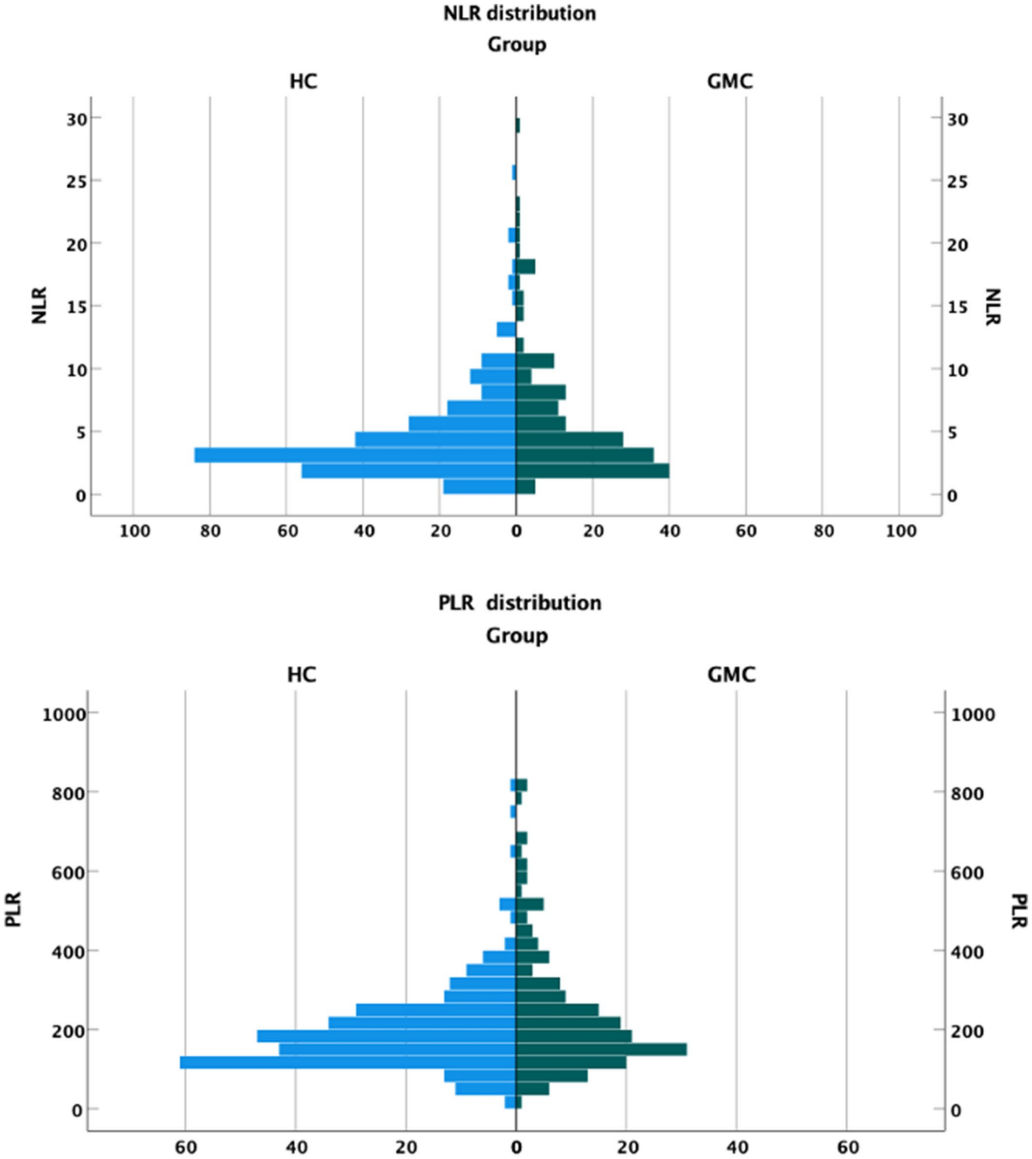
NLR and PLR distribution in each cohort.

Figure 1 presents the distribution of NLR and PLR values in the 2 cohorts. Both NLR and PLR were higher among the GMC patients. The median NLR was 3.95 in the GMC group and 3.54 in the HC group. The median PLR was 191.12 in the GMC group and 175.71 in the HC group. Both differences were statistically significant (Table 4).

**Table 4:** NLR and PLR.

	Shmuel		Shamir		*p*
	*N*	Median	*N*	Median	
NLR	177	3.95 (2.44–7.36)	289	3.54 (2.44–5.56)	0.043
PLR	177	191.12 (137.1–82.2)	289	175.71 (128–237.78)	0.022

## Discussion

This study presents the results of two very different patient groups admitted to COVID-19 departments. The first group consisted of frail, dependent older adults with many comorbidities admitted to COVID-19 departments in a geriatric medical center, with mild or asymptomatic disease. The second group was comprised of younger healthier patients with symptomatic disease requiring hospital admission. The results imply a stronger association between NLR and PLR with age and frailty than with the severity of the disease.

The NLR and PLR were previously described as easily accessible inflammation markers that can help identify COVID severity. Both markers were found to have prognostic value in different medical conditions, such as malignancies, surgical risk, venous thromboembolism and more (2–4, 18, 20, 29–31). Elevated NLR and PLR were described also to rise in frail and older patients (19, 32, 33). Their role in predicting COVID-19 severity had been described in several studies with conflicting results. Seyit et al. (9) have described elevated NLR and PLR in COVID-19 patients. The cohort they described consisted of young patients and the severity was not assessed. Ortega-Rojas et al. (15) conclude that NLR and PLR are predictors of a higher risk of mortality from COVID-19 in older adults. In their work, the subjects had a median age of 70, while most patients had no prior comorbidities, and an extremely high mortality rate was reported. Several other studies described NLR as a predictor of COVID severity and outcomes. In a meta-analysis published in April 2021, NLR was found to present significantly higher levels in advanced COVID-19 stages, showing a good ability to diagnose and predict outcomes (12). Most studies included in the analysis did not stratify according to age or frailty. The outcomes described are in contradiction with the results presented here that show higher NLR in the less severe COVID-19 patients.

Lian et al. (10) describe another cohort of older adults where NLR was independently associated with a progression to critical illness. It is unclear whether these patients were frail, as most did not suffer from significant comorbidities. Age and comorbidities also significantly affected outcomes in the study. Another study of older frailer patients by Olivieri et al. (16) found NLR and PLR to be predictors of in-hospital mortality, independent of age, gender, and other potential confounders.

Our study is unique due to the two poles presented by the different cohorts. The results imply a stronger association of NLR and PLR to older age, frailty and comorbidities rather than to COVID severity.

This study has several limitations. The information on the outcomes is missing, making it difficult to discuss outcomes, such as mortality. Frailty was not assessed in the HC cohort. We believe the presented major differences between the cohorts make it reasonable to assume that the GMC cohort was more frail. The length of stay was longer in the GMC cohort, which is probably the result of the regulatory rules for obligatory isolation at the time of the study. Some patients in the HC group were presumably discharged to complete isolation at home, while the GMC patients were unable to do so.

It is possible that NLR and PLR play a prognostic role after stratification according to age and frailty. Such stratification in older adults requires further large-scale studies. Meanwhile using NLR and PLR as simple prognostic markers in older adults with COVID-19 should be done cautiously.

Finally, the large differences in NLR and PLR may be driven partially by the differences in comorbidities between the groups. Most prior studies describe NLR and PLR at acute rather than chronic medical conditions (5–7, 20, 30, 31, 34–37). Furthermore most COVID-19 patients who are at high risk for developing severe illness are older patients with comorbidities (23, 38–43). This emphasizes again the need for stratification of NLR and PLR before they can be routinely used for prognostic purposes.

## Conclusion

NLR and PLR seem to be affected more by age and frailty than by COVID-19 severity. Their use as prognostic factors for COVID severity should be considered cautiously and should be stratified according to age, frailty and comorbidities. Further research is needed to find whether these markers have a predictive ability in certain age groups or according to frailty status. The results of this study call for further research on NLR and PLR roles as markers of frailty.

## Data availability statement

The original contributions presented in the study are included in the article/supplementary material, further inquiries can be directed to the corresponding author.

## Ethics statement

The studies involving human participants were reviewed and approved by local institutional review and conforms to the principles outlined in the Declaration of Helsinki (IRB Asf0071-21). Due to the study’s retrospective nature, informed consent was wavered by the ethic’s committee. Written informed consent for participation was not required for this study in accordance with the national legislation and the institutional requirements.

## Author contributions

YL: conceptualization, methodology, and writing – original draft. ED: formal analysis and writing – review & editing. AS: conceptualization and investigation. DK: methodology and investigation. AD: investigation. VC: investigation and validation. IS: writing – review & editing. NK: conceptualization, methodology, validation and writing – reviewing and editing, supervision. All authors contributed to the article and approved the submitted version.

## Conflict of interest

The authors declare that the research was conducted in the absence of any commercial or financial relationships that could be construed as a potential conflict of interest.

## Publisher’s note

All claims expressed in this article are solely those of the authors and do not necessarily represent those of their affiliated organizations, or those of the publisher, the editors and the reviewers. Any product that may be evaluated in this article, or claim that may be made by its manufacturer, is not guaranteed or endorsed by the publisher.
